# Editorial: Rising stars in PET and SPECT: 2024

**DOI:** 10.3389/fnume.2025.1723945

**Published:** 2025-11-03

**Authors:** Mario Petretta

**Affiliations:** University of Naples Federico II, Naples, Italy

**Keywords:** PET, SPECT, radionuclide imaging, diagnosis, prognosis

**Editorial on the Research Topic**
Rising stars in PET and SPECT: 2024

It is a great pleasure for me to present in this issue of Frontiers in Nuclear Medicine the Research Topic *Rising Stars in PET and SPECT: 2024*. I would first like to take this opportunity to thank those who responded to the invitation to submit articles and the reviewers whose comments and suggestions helped to improve the quality of the Research Topic. The articles in this Topic were written by Rising Stars researchers in the field of Nuclear Medicine, selected by the Frontiers in Nuclear Medicine editors according to their potential influence on future directions in their respective fields. We hope that these talented young researchers in the field of PET and SPECT will shine a light to guide our future and our path to excellence. The works presented in this Research Topic cover various fields of PET and SPECT and describe theoretical, methodological, and practical advances in issues of clinical interest. Indeed, the Research Topic cover an interesting variety of issues, such as:

1. The likely usefulness of performing V/Q-SPECT/CT imaging in patients presenting with respiratory deterioration following endoscopic lung volume reduction;
2. The central role of FAPI PET/CT for the work-up and management of lymphoma patients;
3. The potential of the Self-SiMilARiTy-Aware Generative Adversarial Framework (SMART) in low count PET images (SMART-PET) to synthesize standard of care activity PET images;
4. The feasibility of ^68^Ga-Trivehexin for imaging of αvβ6-integrin expression in pancreatic cancer and its ability to distinguish primary carcinoma and metastases from background tissue.

The aim of this Editorial is to offer the reader a brief presentation of the articles in this Research Topic, mentioning the value that the individual contributions bring to the horizons and results of research in the field of PET and SPECT. This brief presentation hopes to encourage the reader to delve deeper into the individual contributions, which contribute to the promise of a future of innovation and progress.

The paper by Quartuccio et al. explores the role of FAPI PET/CT in lymphoma patients. In particular, while fluorodeoxyglucose PET/CT (FDG PET/CT) is generally considered the gold standard imaging technique for the initial evaluation and follow-up of lymphoma patients, it is not uncommon for this approach to prove inconclusive. Therefore, fibroblast activation protein inhibitor PET/CT (FAPI PET/CT) has been widely explored as a useful resource. The authors therefore performed a thorough systematic review of the literature available on PubMed/MEDLINE and Cochrane CENTRAL of studies with FAPI PET/CT in lymphoma patients according to the QUADAS-2 criteria. The systematic review reveals that FAPI PET/CT exhibits lower diagnostic sensitivity than [^18^F]-fluorodeoxyglucose (^18^F-FDG) PET/CT in lymphomas characterized by low FAP expression. Nevertheless, FAPI PET/CT retains potential as a complementary imaging modality. In particular, it could help identify lymphoma subgroups with distinct stromal environments, potentially serving as a prognostic biomarker.

The article by Raymond et al. present a novel PET only deep learning framework, the Self-SiMilARiTy-Aware Generative Adversarial Framework (SMART), which leverages Generative Adversarial Networks (GANs) and a self-similarity-aware attention mechanism for denoising ^18^F-FDG PET images. This approach was developed with the aim of obtaining PET images with high diagnostic quality while minimizing the radiation risk. In fact, minimizing radiation risks while preserving PET image quality could potentially enlarge the current applications of PET medical imaging, in particular for longitudinal evaluations and in radiosensitive populations such as pediatrics. This solution can be implemented by denoising PET images with low injected activity. However, the proposed method differs from previous algorithms that rely on structural or anatomical guidance from magnetic resonance imaging (MRI) and fails to effectively preserve global spatial features in denoised PET images, without impacting signal-to-noise ratios. The results of the study indicate that SMART-PET shows promise in reducing noise in PET images and can synthesize diagnostic quality images with a 90% reduction in standard of care injected activity.

The paper by Rhem et al. aimed to determine the biokinetics, image contrast, and acquisition parameters for ^68^Ga-Trivehexin PET imaging in pancreatic cancers. This technique uses the radiopharmaceutical ^68^Ga-Trivehexin to visualize tissues that overexpress integrin αvβ6, particularly tumors. This integrin is part of a family of cell membrane receptors, primarily present in epithelial cells and highly expressed in various tumors, making them a promising target for therapy and diagnosis. Integrins are heterodimeric transmembrane receptors (composed of an alpha and a beta subunit) present on the cell surface that bind the extracellular matrix and possibly other components such as viral or bacterial proteins, and other cells. Serving as a link between the outside and inside of the cell, they transmit bidirectional signals that influence cell motility, growth, differentiation, and survival. In addition to their physiological role, they are also crucial in pathological conditions such as cancer and inflammatory diseases. Integrin αvβ6, integrin αvβ6 is involved in tumor progression, migration, and invasion. Furthermore, integrin αvβ6 molecules can be transferred to other cells via extracellular vesicles. ^68^Ga-Trivehexin PET imaging involves intravenous injection of the tracer and acquisition of PET/CT images after 45–60 min to detect the distribution of the radioisotope in the body, allowing 3D visualization of radioactivity and identification of tumor areas. The results of the study by Rhem et al. indicate that ^68^Ga-Trivehexin is suitable for imaging of αvβ6-integrin expression in pancreatic cancer due to its ability to distinguish primary carcinoma and metastases from background tissue.

Kamga et al. present a case where a paradoxical intrapulmonary shunt was detected several months after treatment. The report is interesting in that it outlines a late paradoxical complication of endoscopic lung volume reduction, a less invasive alternative to lung volume reduction surgery in the treatment of patients with severe emphysema. Indeed, although early complications have been documented, little information is available regarding late paradoxical phenomena.

In conclusions, the articles selected for this Research Topic highlight the potential of advances in nuclear medicine to improve the diagnostic and therapeutic workup of diseases. Increasingly accurate imaging techniques, artificial intelligence algorithms, and innovative radiotracers are expanding the boundaries and indications of nuclear medical imaging. It should be emphasized, however, that biomedical knowledge is constantly evolving, as are epidemiological scenarios. Therefore, increased research collaboration and full sharing of results across different fields are essential to address these challenges and realize the full potential of various proposed innovative approaches. Our hope is that these articles will contribute to this goal and encourage young talents to continue their efforts to advance our knowledge in outcome research and implement the results obtained in daily clinical practice.

